# Mannose-binding lectin-associated serine protease 2 (MASP-2) contributes to poor disease outcome in humans and mice with pneumococcal meningitis

**DOI:** 10.1186/s12974-016-0770-9

**Published:** 2017-01-03

**Authors:** E. Soemirien Kasanmoentalib, Mercedes Valls Seron, Bart Ferwerda, Michael W. Tanck, Aeilko H. Zwinderman, Frank Baas, Arie van der Ende, Matthijs C. Brouwer, Diederik van de Beek

**Affiliations:** 1 Department of Neurology, Academic Medical Center, Amsterdam Neuroscience, Amsterdam, The Netherlands; 2 Department of Clinical Epidemiology, Biostatistics, and Bioinformatics, Academic Medical Center, University of Amsterdam, Amsterdam, The Netherlands; 3 Department of Genome Analysis, Academic Medical Center, Amsterdam, The Netherlands; 4 Department of Medical Microbiology, Center of Infection and Immunity Amsterdam (CINIMA), Academic Medical Center, Amsterdam, The Netherlands; 5 The Netherlands Reference Laboratory for Bacterial Meningitis, Center of Infection and Immunity Amsterdam (CINIMA), Academic Medical Center, Amsterdam, The Netherlands; 6 Department of Neurology, Academic Medical Center, University of Amsterdam, Amsterdam Neuroscience, PO Box 22660, 1100 DD Amsterdam, The Netherlands

## Abstract

**Background:**

Pneumococcal meningitis is the most common and severe form of bacterial meningitis. Fatality rates are substantial, and long-term sequelae develop in about half of survivors. Disease outcome has been related to the severity of the pro-inflammatory response in the subarachnoid space. The complement system, which mediates key inflammatory processes, has been implicated as a modulator of pneumococcal meningitis disease severity in animal studies.

**Methods:**

We investigated mannose-binding lectin-associated serine protease (MASP-2) levels in cerebrospinal fluid (CSF) samples derived from the diagnostic lumbar puncture, which was available for 307 of 792 pneumococcal meningitis episodes included in our prospective nationwide cohort study (39%), and the association between these levels and clinical outcome. Subsequently, we studied the role of MASP-2 in our experimental pneumococcal meningitis mouse model using *Masp2* ^*−/−*^ mice and evaluated the potential of adjuvant treatment with MASP-2-specific monoclonal antibodies in wild-type (WT) mice.

**Results:**

MASP-2 levels in cerebrospinal fluid of patients with bacterial meningitis were correlated with poor functional outcome. Consistent with these human data, *Masp2*-deficient mice with pneumococcal meningitis had lower cytokine levels and increased survival compared to WT mice. Adjuvant treatment with MASP-2-specific monoclonal antibodies led to reduced complement activation and decreased disease severity.

**Conclusions:**

MASP-2 contributes to poor disease outcome in human and mice with pneumococcal meningitis. MASP-2-specific monoclonal antibodies can be used to attenuate the inflammatory response in pneumococcal meningitis.

**Electronic supplementary material:**

The online version of this article (doi:10.1186/s12974-016-0770-9) contains supplementary material, which is available to authorized users.

## Background

Acute community-acquired bacterial meningitis is a life-threatening disease associated with substantial morbidity and mortality and ranks among the top ten infectious causes of death [1]. *Streptococcus pneumoniae* is the most common cause of bacterial meningitis in adults accounting for 50–70% of cases in developed countries [2, 3]. Pneumococcal meningitis is associated with a mortality ranging from 19 to 37%, and neurological sequelae such as hearing loss, focal deficits, and motor and cognitive impairments significantly affect the quality of life of survivors [2, 4–6]. Predisposing factors for pneumococcal meningitis include pneumonia, otitis, sinusitis, cerebrospinal fluid (CSF) leaks, splenectomy or asplenic states, and primary or acquired immune deficiencies [3, 5]. Genetic studies of extreme phenotypes have revealed that patients with single-gene inborn errors in MyD88, IRAK4, and NEMO affecting the activation of the canonical Toll-like receptor (TLR) and interleukin (IL)-1R signaling pathways or in complement components are susceptible for invasive pneumococcal disease [7–10].

Experimental studies in pneumococcal meningitis showed that the host inflammatory response also contributes to an adverse outcome [11]. The initial host inflammatory response is activated by recognition of pathogen-associated molecular patterns by the innate immune system, e.g., by Toll-like receptors and inflammasomes [11]. Subsequently, the complement system is activated, leading to massive release of anaphylotoxins and chemotaxis and activation of neutrophils [11]. Genetic variants in complement system, TLR and IL-1R signaling pathways, and the M-TOR pathway have been identified to be associated with outcome in pneumococcal meningitis [7, 9, 12–15]. Inhibition of the final common pathway in the complement cascade has been identified as target for adjunctive treatment for experimental pneumococcal meningitis. Complement component 5 (C5) inhibition was shown to reduce inflammation of the central nervous system and improve outcome [13, 16]. C5 inhibition shuts down the terminal complement effector pathway but does not prevent the release of anaphylatoxin C3a and other upstream complement activation events. Therefore, inhibition of the upstream activation pathways of the complement system may result in a more complete reduction of complement-mediated inflammation.

The classical pathway was initially proposed as the predominant pathway responsible for complement activation during pneumococcal infection [17]. C1q-deficient mice in which the classical pathway is disrupted and factor B-deficient mice with a defective alternative pathway were both more susceptible to intranasal and intraperitoneal pneumococcal infection compared to wild-type mice. Mice deficient in complement factor 3, affecting all pathways of complement activation, had increased susceptibility to invasive pneumococcal infection, as compared to C1q, factor B, and C1q/FactorB knockouts, suggesting an additional and important role of the lectin pathway in pneumococcal infection [17]. This was confirmed by a study in *Masp2*-deficient mice that showed increased susceptibility to intranasal pneumococcal infection due to decreased opsonization of *S. pneumoniae* [18]. *Masp2*-deficient mice had similar survival times and mortality rates compared to those previously reported in C1q-deficient mice [18].

Thus, an intact complement system protects against contraction of pneumococcal infection; however, in view of the detrimental role of inflammation in disease progression, inhibition of mannose-binding lectin-associated serine protease (MASP-2) presents to be an interesting target for complement blockade aiming to decrease inflammation in pneumococcal meningitis. We studied multiple aspects of the role of MASP-2 in pneumococcal meningitis. First, we measured MASP-2 in CSF of pneumococcal meningitis patients. Subsequently, we analyzed the role of MASP-2 during experimental meningitis in our validated pneumococcal meningitis mouse model using *Masp2* knockout mice. Finally, we studied whether treatment with MASP-2 antibodies improved outcome in a randomized controlled investigator-blinded mouse experiment.

## Methods

### Dutch bacterial meningitis cohort

In a nationwide prospective cohort study, we included adults with community-acquired bacterial meningitis with positive CSF cultures who were identified by the Netherlands Reference Laboratory for Bacterial Meningitis (NRLBM). Methods have been described in detail previously [19]. The NRLBM provided the names of the hospitals where patients with bacterial meningitis had been admitted 2 to 6 days previously. The treating physician was contacted, and informed consent was obtained from all participating patients or their legally authorized representatives. Outcome was graded at discharge according to the Glasgow Outcome Scale (GOS), a well-validated instrument [20]. A score of 1 indicates death; a score of 2 indicates a vegetative state; a score of 3 indicates severe disability; a score of 4 indicates moderate disability; and a score of 5 indicates mild or no disability. A favorable outcome was defined as a score of 5 and unfavorable outcome as a score of 1 to 4. The study was approved by the medical ethical committee of the Academic Medical Centre, Amsterdam, the Netherlands.

### CSF measurements

To evaluate whether MASP-2 CSF levels were increased in CSF during pneumococcal meningitis and whether the concentration was related to severity of disease and outcome, we measured MASP-2 CSF levels in patients and controls. CSF of patients was obtained from the diagnostic lumbar puncture. Control samples were derived from leftover CSF of patients with acute headache in whom a lumbar puncture was performed to exclude a subarachnoid hemorrhage. These control CSF samples all had normal leukocyte count and protein and glucose levels; the final diagnosis in these patients was benign thunderclap headache. CSF was centrifuged and the supernatant was stored at −80 °C until assayed. MASP-2, C5a, and C5b-9 levels were determined using commercially available ELISA kits (MyBioSource and Microvue Quidel, respectively) according to manufacturer instructions.

### Pneumococcal meningitis mouse model

To further determine the role of MASP-2 during pneumococcal meningitis and evaluate whether blocking MASP-2 could be used to improve outcome, we used our well-validated pneumococcal meningitis mouse model [21]. In this model, C57BL/6NCrl mice (Charles River Laboratories, Germany), aged 8–12 weeks old, were weighed and clinically examined prior to infection. Bacterial meningitis was induced by intracisternal injection of 1 μl of 10^7^ CFU/ml *S. pneumoniae* serotype 3 (ATCC 6303; American Type Culture Collection, Rockville, MD, USA) under short-term isoflurane anesthesia. Controls were injected with 1 μl sterile saline. All animals were clinically examined and scored directly following inoculation and at regular intervals. Clinical scoring consisted of weight loss, activity, time to return to upright position, state of fur, posture, eye discharge or protrusion, respiration rate, irregular/labored breathing, epilepsy, limb paresis, and coordination. In healthy mice, the score was 0, and mice with a score of 15 or more were critically ill and therefore euthanized. Other humane endpoints were >25% weight loss, ≥2 seizures per 15 min, status epilepticus, and hemi-paralysis. Mice were euthanized by intraperitoneal injection of ketamine (190 mg/kg, Eurovet Animal Health, Bladel, the Netherlands) in combination with dexmedetomidine (0.3 mg/kg, Pfizer Animal Health, Capelle aan den IJssel, the Netherlands). After, euthanasia blood was collected by transcardial puncture and citrated in a 1:4 citrate to blood ratio. CSF was collected by puncture of the cisterna magna and diluted 1:100 in sterile saline. Subsequently, mice were perfused with sterile phosphate-buffered saline and the brain, spleen, and lung were harvested. The right hemisphere was snap frozen in liquid nitrogen, and the left hemisphere, spleen, and lung were taken up in 20% weight per volume sterile saline and homogenized with a tissue homogenizer. Bacterial titers were determined by plating serial tenfold dilutions of blood, CSF, brain, spleen, and lung homogenates on sheep-blood agar plates and incubating for 16 h at 37 °C. Citrated blood was centrifuged at 2000 rpm for 15 min at 4 °C. Tissue homogenates were lysed as described before [21]. Plasma, CSF, and lysed supernatant were stored at −20 °C until assayed. Experiments were approved by the Institutional Animal Care and Use Committee of the Academic Medical Center, Amsterdam, the Netherlands.

### MASP-2 expression experiments

To evaluate the expression profile of MASP-2, pneumococcal meningitis was induced and mice were sacrificed at 6 h (*n* = 5), 24 h (*n* = 5), and 48 h (*n* = 5) after infection. Mice inoculated with sterile saline and sacrificed at 24 h (*n* = 5) served as control. Mice in the 48 h group were treated intraperitoneal with ceftriaxone (100 mg/kg) at 20 h after infection.

### *Masp2* deficiency experiments

To evaluate the effect of *Masp2* deficiency on disease severity and mortality, C57BL/6NCrl wild-type (WT) mice (*n* = 12) and *Masp2*-deficient mice with a C57/BL6N background (*Masp2* ^*−/−*^, Omeros Corp., *n* = 12) were infected and observed for 60 h in a survival experiment. Mice deficient in *Masp2* have been described elsewhere [22]. In a time point experiment, mice were euthanized at 6 (WT infected *n* = 12, *Masp2* ^*−/−*^ infected *n* = 12) and 30 (WT infected *n* = 12, *Masp2* ^*−/−*^ infected *n* = 12, WT control *n* = 6, *Masp2* ^*−/−*^ control *n* = 6) h after infection.

### Adjuvant treatment with MASP-2 antibodies

The previously described recombinant monoclonal antibody AbD04211 inhibits mouse MASP-2 and is able to block lectin pathway activity for 7 days when given intraperitoneally in a single dose of 0.6 mg/kg [22]. To assess the effect of adjuvant treatment with this antibody against MASP-2 on disease severity and mortality, a survival study was performed. WT mice were infected and treated intraperitoneally at 20 h after infection with ceftriaxone (100 mg/kg) in combination with adjuvant treatment and observed for 68 h. Adjuvant treatment consisted of sterile saline (*n* = 24), isotype antibodies (MAB205P, 1 mg/kg, *n* = 24), or MASP-2 antibodies (AbD04211, 1 mg/kg, *n* = 24). When the sample size in each group is 24, a 0.05 level two-sided log-rank test for equality of survival curves will have 80% power to detect a decrease in mortality from 45 and 10%. In a second experiment, mice were treated with adjuvant sterile saline (*n* = 18) or MASP-2 antibodies (AbD04211, 7 mg/kg, *n* = 18) and euthanized at 24 and 48 h after infection.

### Protein expression

IL-1β, IL-6, IL-10, keratinocyte chemoattractant (KC), tumor necrosis factor (TNF)-α, and macrophage inflammatory protein (MIP)-2 levels were determined in mouse plasma and brain homogenates with Luminex® technology (Bio-Rad Laboratories). Expression of MASP-2 and C5b-9 was measured in mouse brain homogenates by ELISA (CUSABIO and USCN Life Science, respectively). Albumin concentrations in brain homogenates were determined with ELISA (ALPCO Diagnostics).

### Statistics

Continuous variables were compared using the Mann-Whitney *U* test, and dichotomous variables were compared using the chi-squared test. Survival was analyzed using a log-rank test. Clinical scores were compared using linear mixed models with group/treatment, time, and their interaction as effects. For all models, a random slope was modeled and estimates were corrected for autocorrelation and/or unequal variances where appropriate. For all analyses, a *P* value <0.05 was regarded as significant.

## Results

### Cerebrospinal fluid levels of MASP-2 in patients with pneumococcal meningitis

**Fig. 1:**
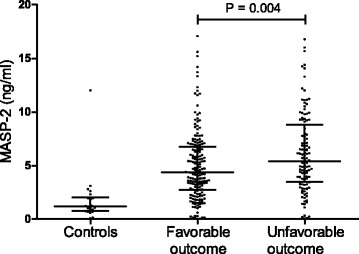
Cerebrospinal fluid MASP-2 concentration and outcome in patients with pneumococcal meningitis. The MASP-2 concentration was significantly higher in patients with an unfavorable outcome (*n* = 121) compared to that in patients with a favorable outcome (*n* = 186). Each *dot* represents an individual patient, *lines* represent median values, and *error bars* are interquartile ranges. *P* value was determined with the Mann-Whitney *U* test

In our nationwide prospective cohort study, 792 episodes of community-acquired pneumococcal meningitis were included between March 2006 and May 2012. We investigated MASP-2 levels in cerebrospinal fluid (CSF) samples derived from the diagnostic lumbar puncture, which was available for 307 of 792 pneumococcal meningitis episodes (39%). Baseline characteristics were similar in patients with and without CSF available (Table 1). We also measured MASP-2 CSF concentrations in 24 controls consisting of patients diagnosed with thunderclap headache in whom lumbar puncture was performed to exclude subarachnoid headache. All controls had normal CSF leukocyte count and glucose and protein levels. We found that pneumococcal meningitis patients had increased levels of MASP-2 in their CSF compared to controls (median 4.77 vs. 1.19 ng/ml, *P* < 0.0001). The CSF MASP-2 concentration was significantly higher in patients with an unfavorable outcome, defined as a score of 1 through 4 on the Glasgow Outcome Scale (GOS) [20] compared to patients with a favorable outcome (5.39 vs. 4.36 ng/ml, *P* = 0.004; Fig. 1). CSF MASP-2 concentration was higher in deceased patients compared to that in survivors, but this did not reach statistical significance (5.52 vs. 4.71 ng/ml, *P* = 0.090). In patients with pneumococcal meningitis, CSF levels of MASP-2 were positively correlated to C5a (*ρ* = 0.481, *P* < 0.0001), C5b-9 (*ρ* = 0.560, *P* < 0.0001) and the CSF protein concentration (*ρ* = 0.488, *P* < 0.0001), but not to CSF leukocyte count (*ρ* = 0.088, *P* = 0.130). Genetic variations in the *MASP-2* gene in patients with pneumococcal meningitis did not influence disease severity (Additional file 1).

**Table 1:** Clinical characteristics of 792 pneumococcal meningitis patients with and without CSF available

Characteristics	Patients with CSF (*n* = 307)	Patients without CSF (*n* = 485)	*P* value
Age (year)	62 (51–70)	61 (51–70)	0.96
Male	142/307 (46)	242/458 (53)	0.32
Duration of symptoms <24 h	140/297 (47)	233/464 (50)	0.41
Predisposing conditions	202/307 (66)	318/485 (66)	0.95
Otitis or sinusitis	121/306 (40)	205/483 (42)	0.42
Pneumonia	28/298 (9)	53/461 (11)	0.36
Immunocompromised	96/307 (31)	119/485 (25)	0.04
Symptoms and signs on presentation	^a^	^d^	
Headache	221/267 (83)	345/415 (83)	0.90
Neck stiffness	224/297 (75)	340/447 (76)	0.84
Systolic blood pressure (mmHg)	148 (130–169)	145 (126–165)	0.29
Heart rate (bpm)	100 (86–112)	100 (88–115)	0.78
Body temperature (°C)	39.0 (38.2–39.7)	39.0 (38.0–39.7)	0.26
Score on Glascow Coma Scale	10 (8–13)^b^	10 (9–13)^e^	0.70
<8 indicating coma	40/306 (13)	80/483 (17)	0.18
Focal neurologic deficits	90/306 (29)	139/481 (29)	0.88
Indexes of CSF inflammation	^c^	^f^	
Opening pressure	37 (27–43)	35 (28–45)	0.66
White blood cell count (/mm^3^)	2560 (460–6667)	2567 (516–7048)	0.60
White blood cell count <1000/mm^3^	103/295 (35)	153/465 (33)	0.57
Protein (g/l)	4.1 (2.5–6.0)	4.2 (2.5–6.2)	0.61
CSF blood glucose ratio	0.02 (0.00–0.23)	0.01 (0.00–0.16)	0.06
Positive blood culture	215/267 (81)	341/423 (81)	0.98
Score on Glasgow Outcome Scale			
1—death	43/307 (14)	101/485 (21)	0.02
2—vegetative state	0/307 (0)	1/485 (0)	0.43
3—severe disability	14/307 (5)	27/485 (6)	0.53
4—moderate disability	64/307 (21)	77/485 (16)	0.08
5—good recovery	186/307 (61)	279/485 (58)	0.39

Data are number/number evaluated (percentage), and continuous data are mean ± SD

^a^Systolic blood pressure was evaluated in 301 patients, heart rate was evaluated in 298 patients, and temperature was evaluated in 304 patients

^b^Score on Glasgow Coma Scale was evaluated in 306 patients

^c^CSF opening pressure was evaluated in 76 patients, CSF white blood cell count was evaluated in 295 patients, CSF protein was evaluated in 293 patients, and CSF blood glucose ratio was evaluated in 288 patients

^d^Systolic blood pressure was evaluated in 477 patients, heart rate was evaluated in 472 patients, and temperature was evaluated in 478 patients

^e^Score on Glasgow Coma Scale was evaluated in 483 patients

^f^CSF opening pressure was evaluated in 89 patients, CSF white blood cell count was evaluated in 464 patients, CSF protein was evaluated in 464 patients, and CSF blood glucose ratio was evaluated in 456 patients

### MASP-2 expression in experimental pneumococcal meningitis

To study whether MASP-2 was expressed in our pneumococcal meningitis mouse model [21], WT mice were injected into the cisterna magna with *S. pneumoniae* serotype 3 (ATCC 6303; *n* = 15) or sterile saline (*n* = 5) and sacrificed at 6, 24, or 48 h (*n* = 5 per time point). One mouse reached a humane endpoint before the 48 h time point, and MASP-2 concentrations were determined in brain homogenates of the remaining 19 mice. Mice with pneumococcal meningitis showed increased levels of MASP-2 in brain at 6 (median 2.76 μg/mg tissue, *P* = 0.008) and 24 (median 3.01 μg/mg tissue, *P* = 0.008) h after infection compared to saline-inoculated mice at 24 h after inoculation (median 1.43 μg/mg tissue), but no difference was observed after 48 h (median 1.31 μg/mg tissue).

### *Masp2* deficiency in experimental pneumococcal meningitis

**Fig. 2:**
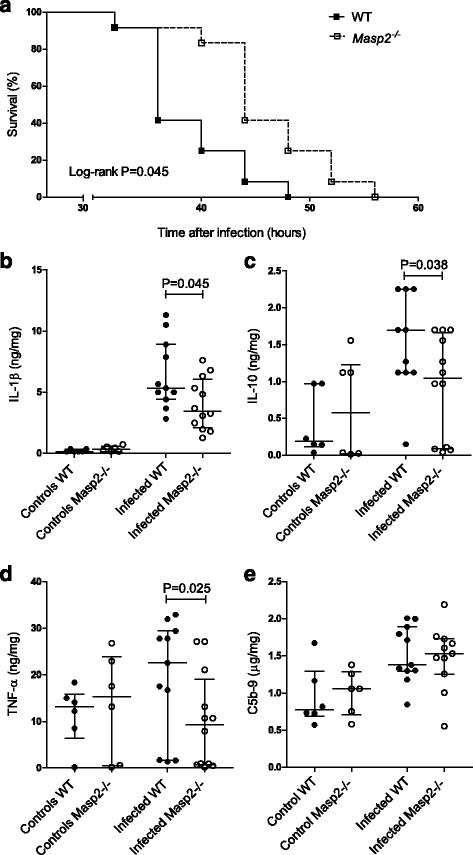
Functional role of MASP-2 in pneumococcal meningitis mouse model. Kaplan-Meier curve showing increased survival in *Masp2* ^*−/−*^ mice during pneumococcal meningitis (**a**). Cytokines and complement levels were measured in brain. *Masp2* ^*−/−*^ mice had significantly lower brain levels of IL-1β, IL-10, and TNF-α 30 h after infection (**b**–**d**). Brain levels of C5b-9 were similar between *Masp2* ^*−/−*^ and WT mice (**e**). Data are given as medians and 75th quartile; *P* values were determined with the Mann-Whitney *U* test

To investigate the role of MASP-2 in disease progression in pneumococcal meningitis, we infected *Masp2* ^*−/−*^ (*n* = 12) and WT (*n* = 12) mice by intracisternal inoculation with *S. pneumoniae* serotype 3 (ATCC 6303) [21]. At 20 h after infection, all mice showed signs of illness, and the first animals reached a humane endpoint at 32 h after infection. All mice reached an endpoint during the 60-h observation period. *Masp2* ^*−/−*^ mice had a significantly longer survival time compared to WT mice (median survival 44 vs. 36 h, log-rank *P* = 0.005; Fig. 2a). Clinical severity scores for *Masp2*-deficient mice increased slower as compared to those for WT mice (0.35 vs. 0.42 points/h, *P* = 0.013).

Next, we infected *Masp2* ^*−/−*^ (*n* = 24) and WT (*n* = 24) mice by intracisternal inoculation with *S. pneumoniae* and euthanized them at 6 and 30 h after infection. Mice inoculated with sterile saline and euthanized at 30 h served as control (*Masp2* ^*−/−*^ *n* = 6 and WT *n* = 6). One WT mice with pneumococcal meningitis reached an endpoint before the 30-h time point. Bacterial outgrowth in CSF, blood, brain, spleen, and lung was similar between *Masp2* ^*−/−*^ and WT mice at 6 and 30 h after infection (Additional file 1: Figure S1). *Masp2* ^*−/−*^ mice had significantly lower brain levels of interleukin (IL)-1β (median 3.46 vs. 5.33 ng/mg tissue, *P* = 0.045), IL-10 (1.04 vs. 1.70 ng/mg tissue, *P* = 0.038), and TNF-α (9.32 vs. 22.58 ng/mg tissue, *P* = 0.025) compared to WT mice at 30 h after infection (Fig. 2b–d). No differences were observed between *Masp2* ^*−/−*^ and WT mice in brain IL-6, KC, and MIP-2 levels or plasma levels of IL-1β, IL-6, IL-10, KC, TNF-α, and MIP-2. Complement activation, as indicated by brain levels of C5b-9, was also similar between *Masp2* ^*−/−*^ and WT mice 30 h post-infection (*P* = 0.88; Fig. 2e). During bacterial meningitis, destruction of the blood-brain barrier is related to disease progression and is reflected by increased brain albumin levels [11]. Brain albumin content was elevated in *Masp2* ^*−/−*^ (312.23 vs. 86.27 μg/mg tissue, *P* = 0.006) and WT (262.21 vs. 42.85 μg/mg tissue, *P* < 0.001) mice at 30 h after infection compared to saline-inoculated mice. No differences were observed between *Masp2* ^*−/−*^ and WT mice at 6 (*P* = 0.80) and 30 (*P* = 0.74) h post-infection.

### Adjuvant treatment with MASP-2 antibodies

We compared the effect of inhibitory MASP-2 antibodies with saline in our treatment model. Thirty-six WT mice were infected intracisternally with *S. pneumoniae* serotype 3 (ATCC 6303). At 20 h post-infection, all mice were treated with ceftriaxone intraperitoneally plus either MASP-2 antibodies (*n* = 18) or saline (*n* = 18) and euthanized at 24 and 48 h. MASP-2 antibody-treated mice had reduced brain levels of C5b-9 compared to saline-treated mice at 24 h (median 1.08 vs. 2.53 μg/mg tissue, *P* = 0.017) and 48 h (0.85 vs. 1.25 μg/mg tissue, *P* = 0.052) after infection. Plasma C5b-9 levels were reduced in MASP-2 antibody-treated mice compared to saline-treated mice at 24 h after infection (0.42 vs. 0.63 μg/ml, *P* = 0.022). MASP-2 antibody treatment was associated with lower plasma levels of TNF-α compared to saline at 48 h after infection (median 0.10 vs. 0.25 ng/ml, *P* = 0.044). No differences were observed in bacterial CFUs (Additional file 1: Figure S2), brain levels of IL-1β, IL-6, IL-10, KC, TNF-α, and MIP-2, and plasma levels of IL-1β, IL-6, IL-10, KC, and MIP-2 between MASP-2 antibody- and saline-treated mice.

**Fig. 3:**
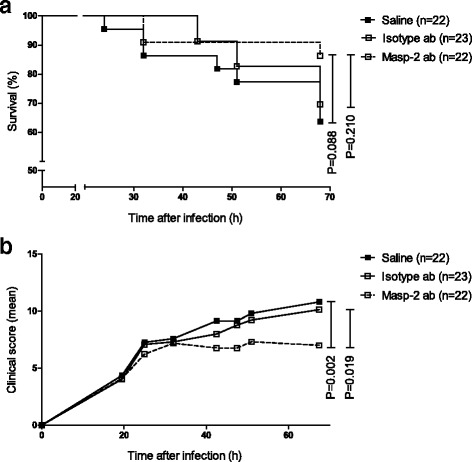
The effect of adjuvant treatment with the MASP-2 antibodies on clinical severity and survival in experimental pneumococcal meningitis. Kaplan-Meier curve of WT mice with pneumococcal meningitis treated intraperitoneally 20 h after infection with ceftriaxone (100 mg/kg) in combination with adjuvant treatment and observed for 68 h (**a**). Adjuvant treatment consisted of sterile saline, isotype antibodies (MAB205P, 1 mg/kg), or MASP-2 antibodies (D04211, 1 mg/kg). There was no difference in survival between groups. *P* values were determined with the log-rank test. Clinical severity scores for MASP-2 antibody-treated mice increased slower as compared to saline- (0.017 vs. 0.103 points/h) and isotype antibody (0.0017 vs. 0.080 points/h)-treated mice (**b**). *P* values were determined using linear mixed models with group/treatment, time, and their interaction as effects

Next, we evaluated the effect of adjuvant treatment with the MASP-2 antibodies on clinical severity and survival. Pneumococcal meningitis was induced in 72 WT mice; three mice showed a limb paresis directly following inoculation, and two mice reached a humane endpoint before treatment was given and were excluded from the experiment. The remaining 67 mice all showed signs of infection 20 h post-infection and were randomized for antibiotic therapy consisting of ceftriaxone intraperitoneally in combination with either saline (*n* = 22), isotype antibodies (*n* = 23), or MASP-2 antibodies (*n* = 22). The 68-h mortality rates were 8 of 22 (36%) in the saline group, 7 of 23 (30%) in the isotype antibody group, and 3 of 22 (14%) in the MASP-2 antibody group (Fig. 3a; Fisher’s exact test for overall difference *P* = 0.21). Mortality in the MASP-2 antibody-treated group was lower compared to sham treatment, but this did not reach statistical significance (MASP-2 Ab vs. saline *P* = 0.088; MASP-2 Ab vs. isotype antibody *P* = 0.210). Clinical severity scores for MASP-2 antibody-treated mice increased significantly slower as compared to saline (Fig. 3b; 0.017 vs. 0.103 points/h, *P* = 0.002) and isotype antibody-treated mice (0.0017 vs. 0.080 points/h, *P* = 0.019).

## Discussion

Our results show that MASP-2 contributes to severity and outcome in pneumococcal meningitis. In patients with pneumococcal meningitis, MASP-2 concentration was elevated in the CSF and high levels were associated with poor functional outcome. In experimental pneumococcal meningitis, *Masp2* deficiency led to decreased disease severity through decreased brain inflammation. Adjuvant treatment with MASP-2-specific monoclonal antibodies led to a decreased level of complement activation and disease severity.

The identified role of MASP-2 is consistent with the two-edged sword theory on complement activation in infectious disease [23]. A less readily activated complement system leads to increased susceptibility to infection but reduced severity of inflammation, which is the key pathogenic mechanism in pneumococcal meningitis. A more readily activated complement system reduces the risk of infection, but results in a poor prognosis in bacterial meningitis [13, 24]. *Masp2* deficiency was previously shown to increase the risk of infection but we now show this leads to a reduced inflammatory response during meningitis, thereby improving survival.

Previous studies in pneumococcal infection showed increased disease severity among *Masp2*-deficient and MASP-2 antibody-treated mice, which was mainly driven by decreased opsonization of bacteria, leading to increased bacterial counts in the peripheral blood [18]. In our study, neither *Masp2* deficiency nor MASP-2 antibody treatment influenced bacterial counts in the blood or brain, and the effect on disease severity seemed to be driven by a decreased inflammatory response. In pneumococcal meningitis, complement activation, notably in the final common pathway, has previously been found to cause brain damage and was associated with an adverse outcome [11, 25]. Reduction of complement activation in C3-deficient mice resulted in less intracranial complications in pneumococcal meningitis, but the lack of C3-mediated opsonophagocytosis led to decreased bacterial clearance and worsened outcome [26]. We hypothesize that in case of *Masp2* deficiency, C3b, formed via the classical or alternative pathway, is still able to opsonize the bacteria for phagocytosis. This is supported by similar levels of C5b-9 in brains of *Masp2*-deficient and WT mice as an indicator of total complement activation. MASP-2 antibody-treated mice had reduced brain levels of C5b-9 compared to saline-treated infected mice. In our treatment model, the MASP-2 antibody was given in conjunction with antibiotic therapy, while previous studies were performed without antibiotic therapy [18]. Another explanation for the difference in results between these previous studies and our study is the difference in pneumococcal serotypes (serotype 2 vs. 3). The binding of M-ficolin, in association with MASP-2, of *S. pneumoniae* has been shown to be serotype-specific, at least for certain pneumococcal serotypes [27]. Other work showed that binding of *S. pneumoniae* by lectin pathway pattern recognition molecules occurs only with a minority of serotypes and that L-ficolin specifically binds to capsule constituents [28]. Although we found no difference in C5b-9 brain levels between *Masp2*-deficient and WT mice after infection, *Masp2* deficiency was associated with decreased brain cytokine levels and an increased survival. We hypothesize that the difference may be explained by a different pace of complement activation in *Masp2*-deficient mice compared to wild-type mice. *Masp2*-deficient mice could reach a full activation state of the complement system at a later time point, which would explain the decreased inflammatory response and increased survival.

Adjunctive treatment with MASP-2 antibodies resulted in a reduction of disease severity and tended to reduce mortality despite having no effect on either bacterial outgrowth in the CSF and blood or antibiotic-induced bacterial killing in experimental pneumococcal meningitis. In our treatment model, we observed no effect on brain cytokine levels, but there was an effect on total C5b-9 brain levels. The protective effect holds promise for future treatment of patients with pneumococcal meningitis, but the effect size seemed to be smaller than previously observed for adjunctive treatment with anti-C5 antibodies [13, 16]. However, experiments using adjunctive treatment with anti-C5 antibodies were performed in a model of pneumococcal meningitis with high disease severity, with a mortality rate of 100% in the placebo group [16]. C1 inhibitors have been shown to reduce clinical severity, increase bacterial clearance, and decrease CSF and meningeal inflammation in a rat model of pneumococcal meningitis [29]. Altogether, these studies suggest that inhibition of complement activation may be beneficial in pneumococcal meningitis.

Genetic variations in the *Masp2* gene did not explain inter-individual differences in disease course. However, this does not negate the importance of MASP-2 in activation of complement in pneumococcal meningitis. Involvement of the lectin pathway in invasive pneumococcal disease has been shown by genetic association studies on mannose-binding lectin (MBL)2 deficiency [7]. In a prospective genetic association study, including 299 patients with pneumococcal meningitis and 216 controls, we previously showed that the risk for contracting pneumococcal meningitis was substantially increased for white individuals homozygous with the defective MBL2 0/0 genotype (odds ratio 8.21, 95% confidence interval 1.05–64.1; *P* = 0.017) [30]. CSF MBL levels were significantly lower in patients with the A/0 and 0/0 genotype compared to homozygotes with the wild-type alleles (A/A; *P* < 0.001). CSF MBL levels were positively correlated with C3a and iC3b levels, indicating complement activation by the lectin pathway.

Our study has several limitations. First, selection bias could have been introduced since CSF was only available in a portion of patients included in the cohort. However, patients with and without CSF available had similar baseline and clinical characteristics, and therefore, selection bias is unlikely. Second, our analysis of CSF MASP-2 levels and disease severity is associative and cannot be seen as proof of causation. Third, we did not backcross the mice knockout and wild-type strains because of time and cost considerations. Although the animals are from the same genetic background (C57/BL6N), they were not identical except for the *Masp2* gene. This means we cannot rule out that an unidentified small variation between strains may contribute to the identified phenotype. However, this does not influence the CSF and antibody treatment studies, suggesting a similar role of MASP2 as established in the knockout experiments. Fourth, our animal experiments were not performed in a blinded and randomized manner, which may lead to an overestimation of the treatment effect. Although parameters of inflammation and outcome seem to be consistent, this is not according to current standards [31]. Then, randomized controlled experimental trials are needed comparing adjunctive treatment with MASP-2 antibodies, with and without adjunctive dexamethasone therapy. Such experimental trials should be adequately powered to detect differences in mortality, done with meningitis caused by different serotypes, and ideally be performed in different experimental laboratories.

## Conclusions

Our study stresses the important role of MASP-2 in pneumococcal meningitis and suggests that MASP-2 inhibitory antibodies can attenuate the harmful inflammatory response during pneumococcal meningitis. Eventually, anti-MASP-2 antibodies may be used for complement inhibition in patients as currently these are studies used in clinical trials (ClinicalTrials.gov Identifier NCT02222545). Although further experimental proof is needed to confirm our results, our results present a promising target for future treatment of pneumococcal meningitis.

## Additional file

Additional file 1: Supplemental Tables 1 and 2, and Figures 1 and 2. (DOC 202 kb)

## Abbreviations

C5: Complement component 5
CSF: Cerebrospinal fluid
IL: Interleukin
KC: Keratinocyte chemoattractant
MASP-2: Mannose-binding lectin-associated serine protease
MBL: Mannose-binding lectin
MIP: Macrophage inflammatory protein
TNF: Tumor necrosis factor
