# Erratum to: Mannose-binding lectin-associated serine protease 2 (MASP-2) contributes to poor disease outcome in humans and mice with pneumococcal meningitis

**DOI:** 10.1186/s12974-017-0857-y

**Published:** 2017-04-06

**Authors:** E. Soemirien Kasanmoentalib, Mercedes Valls Seron, Bart Ferwerda, Michael W. Tanck, Aeilko H. Zwinderman, Frank Baas, Arie van der Ende, William J. Schwaeble, Matthijs C. Brouwer, Diederik van de Beek

**Affiliations:** 1 Department of Neurology, Academic Medical Center, Amsterdam Neuroscience, Amsterdam, The Netherlands; 2 Department of Clinical Epidemiology, Biostatistics, and Bioinformatics, Academic Medical Center, University of Amsterdam, Amsterdam, The Netherlands; 3 Department of Genome Analysis, Academic Medical Center, Amsterdam, The Netherlands; 4 Department of Medical Microbiology, Center of Infection and Immunity Amsterdam (CINIMA), Academic Medical Center, Amsterdam, The Netherlands; 5 The Netherlands Reference Laboratory for Bacterial Meningitis, Center of Infection and Immunity Amsterdam (CINIMA), Academic Medical Center, Amsterdam, The Netherlands; 6 Department of Infection, Immunity and Inflammation, University of Leicester, Leicester, UK; 7 Department of Neurology, Academic Medical Center, University of Amsterdam, Amsterdam Neuroscience, PO Box 22660, 1100 DD Amsterdam, The Netherlands

## Erratum

Upon publication of the original article [1], it was noticed that Prof William J Schwaeble was omitted from the author list. He has now been added in this erratum, along with his affiliation and email address, as a co-corresponding author. Consequently, the numbering of Diederik van de Beek’s affiliation ‘6’ has been shifted to ‘7’.

The new article reference should read:

Kasanmoentalib ES, Valls Seron M, Ferwerda B, Tanck MW, Zwinderman AH, Baas F, van der Ende A, Schwaeble WJ, Brouwer MC, van de Beek D. Mannose-binding lectin-associated serine protease 2 (MASP-2) contributes to poor disease outcome in humans and mice with pneumococcal meningitis. *J Neuroinflammation*. 2017 Jan 3;14(1):2
