# Supplementary material for: Mannose-binding lectin-associated serine protease 2 (MASP-2) contributes to poor disease outcome in humans and mice with pneumococcal meningitis
Source: J Neuroinflammation. 2017 Jan 3;14:2. doi: 10.1186/s12974-016-0770-9 (PMC5234106; doi:10.1186/s12974-016-0770-9)
Supplement: Additional file 1: — Supplemental Tables 1 and 2, and Figures 1 and 2. (DOC 202 kb) [file 12974_2016_770_MOESM1_ESM.doc]

**Additional file 1**

**Nationwide genetic association study** To determine whether genetic variance in *Masp-2* influences outcome of pneumococcal meningitis we analysed *Masp-2* genotypes. Blood from pneumococcal meningitis patients in our nationwide prospective cohort study was collected in sodium/EDTA for DNA extraction. DNA was isolated with the Gentra Puregene Isolation kit (Qiagen) and quality control procedures were performed to determine the yield of isolation. Samples were genotyped using the Illumina Exome array v1.1. Genotyping was performed in collaboration with the Human Genome Facility and the department of Epidemiology, Erasmus MC, the Netherlands as part of the Netherlands ExomeChip Project. Variant calling was conducted using GenomeStudio software, from Illumina, using the default settings. Samples were grouped to approximately 40000 for more accuracy during rare variant SNPs calling. Called data was exported to PLINK to carry out sample and marker quality control (QC) using the protocol described by Anderson et al.[1, 2] Genetic association between groups was tested using the Fisher’s exact test on allelic association as implemented in PLINK.[2] Finally, all markers in the *Masp-2*  gene were extracted and evaluated.

**Results**

Between March 2006 and October 2011 722 episodes of community-acquired pneumococcal meningitis were included in our nationwide prospective cohort study. DNA was available for 474 patients (66%). The *MASP2* gene was genotyped using the Illumina Exome array v1.1. In the analysis all patients of non-European descent were excluded, to prevent the effect of population stratification, resulting in the inclusion of 397 pneumococcal meningitis patients with male/female ratio of 1:1. Predisposing conditions, most commonly otitis media or sinusitis (47%) and immunocompromised state (25%), were present in 68% of episodes (Supplemental Table 1). In 14% of episodes, patients were comatose on admission, and 29% of the episodes had focal neurologic deficits. The case fatality rate was 8%, and 34% of the episodes had an unfavorable outcome, defined as a score of 1 through 4 on the Glasgow Outcome Scale (GOS).[3] Patients for whom DNA was obtained were on average younger and had a better outcome than patients for whom DNA was not obtained (Supplemental Table 2). Subsequently we analysed whether genetic variation in *Masp2* influences disease outcome by comparing patients with a favorable outcome (271 patients) to those with an unfavorable outcome (126 patients). We identified four *Masp2* variations (rs numbers: 2273346, 12711521, 12142107, 139962539) that showed no association with unfavorable outcome (Supplemental Table 3).

**Supplemental Table 1**. **Clinical characteristics of 397 pneumococcal meningitis patients included in the genetic association study.**

| **Characteristics** | **Values** |
| --- | --- |
| Age (yr) | 61 (50-69) |
| Male (no. [%]) | 183 (46) |
| Duration of symptoms < 24 hours | 197/386 (51) |
| Predisposing conditions | 271/397 (68) |
| Otitis or sinusitis | 188/396 (47) |
| Pneumonia | 39/385 (10) |
| Immunocompromised | 101/397 (25) |
| Symptoms and signs on presentation^A^ |  |
| Headache | 302/358 (84) |
| Neck stiffness | 299/382 (78) |
| Systolic blood pressure (mmHg) | 148 (130-169) |
| Heart rate (bpm) | 100 (88-112) |
| Body temperature (°C) | 39.0 (38.2-39.7) |
| Score on Glasgow Coma Scale^B^ | 10 (9-13) |
| <8 indicating coma | 56/397 (14) |
| Focal neurologic deficits | 116/396 (29) |
| Indexes of CSF inflammation^C^ |  |
| Opening pressure | 37 (29-45) |
| White blood cell count (/mm^3^) | 2930 (613-7849) |
| White blood cell count < 1000/mm^3^ | 115/380 (29) |
| Protein (g/l) | 4.1 (2.4-6.2) |
| CSF blood glucose ratio | 0.02 (0.00-0.21) |
| Positive blood culture | 286/359 (80) |
| Score on Glasgow Outcome Scale |  |
| 1 – death | 30/397 (8) |
| 2 – vegetative state | 1/397 (0.3) |
| 3 – severe disability | 19/397 (5) |
| 4 – moderate disability | 83/397 (21) |
| 5 – good recovery | 264/397 (67) |

Data are number/number evaluated (percentage), and continuous data are median (interquartile range). ^A^Systolic blood pressure was evaluated in 394 patients, heart rate was evaluated in 392 patients and temperature was evaluated in 396 patients. ^B^Score on Glasgow Coma Scale was evaluated in all patients. ^C^CSF opening pressure was evaluated in 84 patients, CSF white blood cell count, CSF protein and CSF blood glucose ratio were evaluated in 380 patients.

**Supplemental Table 2**. Clinical characteristics of 722 pneumococcal meningitis patients with and without DNA available.

| **Characteristics** | **Patients with DNA (n=474)** | **Patients without DNA (n=248)** | **p-value** |
| --- | --- | --- | --- |
| Age (yr) | 60 (48-68) | 63 (55-72) | 0.001 |
| Male (no. [%]) | 222/474 (47) | 132/248 (53) | 0.10 |
| Duration of symptoms < 24 hours | 225/461 (49) | 117/236 (50) | 0.85 |
| Predisposing conditions | 323/474 (68) | 151/248 (61) | 0.05 |
| Otitis or sinusitis | 219/473 (46) | 79/247 (32) | <0.001 |
| Pneumonia | 46/459 (10) | 28/235 (12) | 0.44 |
| Immunocompromised | 124/474 (26) | 72/248 (29) | 0.41 |
| Symptoms and signs on presentation | A | D |  |
| Headache | 365/429 (85) | 155/198 (78) | 0.04 |
| Neck stiffness | 357/456 (78) | 158/224 (71) | 0.03 |
| Systolic blood pressure (mmHg) | 146 (130-167) | 143 (125-164) | 0.20 |
| Heart rate (bpm) | 100 (88-112) | 100 (85-116) | 0.72 |
| Body temperature (°C) | 39.0 (38.2-39.7) | 39.0 (37.7-39.6) | 0.14 |
| Score on Glasgow Coma Scale | 10 (9-13)^B^ | 10 (8-13)^E^ | 0.11 |
| <8 indicating coma | 63/474 (13) | 45/248 (18) | 0.08 |
| Focal neurologic deficits | 151/474 (32) | 152/248 (61) | 0.87 |
| Indexes of CSF inflammation | C | F |  |
| Opening pressure | 37 (30-45) | 33 (25-43) | 0.16 |
| White blood cell count (/mm^3^) | 3088 (772-8391) | 2041 (276-5752) | <0.001 |
| White blood cell count < 1000/mm^3^ | 130/456 (27) | 97/237 (39) | 0.001 |
| Protein (g/l) | 4.0 (2.4-6.2) | 4.5 (3.0-6.1) | 0.04 |
| CSF blood glucose ratio | 0.03 (0.00-0.23) | 0.00 (0.00-0.08) | <0.001 |
| Positive blood culture | 340/423 (80) | 178/216 (82) | 0.54 |
| Score on Glasgow Outcome Scale |  |  |  |
| 1 – death | 37/474 (8) | 96/248 (39) | <0.001 |
| 2 – vegetative state | 1/474 (0.2) | 0/248 (0) | 0.47 |
| 3 – severe disability | 23/474 (5) | 13/248 (5) | 0.82 |
| 4 – moderate disability | 90/474 (19) | 43/248 (17) | 0.59 |
| 5 – good recovery | 323/474 (68) | 96/248 (39) | <0.001 |

Data are number/number evaluated (percentage), and continuous data are mean ± SD. ^A^Systolic blood pressure was evaluated in 471 patients, heart rate was evaluated in 468 patients and temperature was evaluated in 473 patients. ^B^Score on Glasgow Coma Scale was evaluated in all patients. ^C^CSF opening pressure was evaluated in 101 patients, CSF white blood cell count was evaluated in 456 patients, CSF protein and CSF blood glucose ratio were evaluated in 454 patients. ^D^Systolic blood pressure was evaluated in 243 patients, heart rate was evaluated in 240 patients and temperature was evaluated in 244 patients. ^E^Score on Glasgow Coma Scale was evaluated in all patients. ^F^CSF opening pressure was evaluated in 54 patients, CSF white blood cell count was evaluated in 236 patients, CSF protein was evaluated in 238 patients and CSF blood glucose ratio was evaluated in 230 patients.

| **SNP^#^** | **Function^$^** | **Alleles** | | **A1 frequency^&^** | | **P-value** |
| --- | --- | --- | --- | --- | --- | --- |
|  |  | **A1** | **A2** | **Unfavorable** | **Favorable** |  |
| rs2273346 | Missense | G | A | 0.028 | 0.018 | 0.433 |
| rs12711521 | Missense | C | A | 0.171 | 0.148 | 0.401 |
| rs12142107 | Synonymous | A | G | 0.024 | 0.017 | 0.576 |
| rs139962539 | Missense | A | G | 0 | 0.011 | 0.185 |

**Supplemental Figure 1.** Bacterial outgrowth in CSF, blood, brain, spleen and lung was similar between *Masp2^-/-^* and WT mice at 6 and 30 hours after infection. Data are given as medians and 75th quartile, P values were determined with the Mann-Whitney U test.


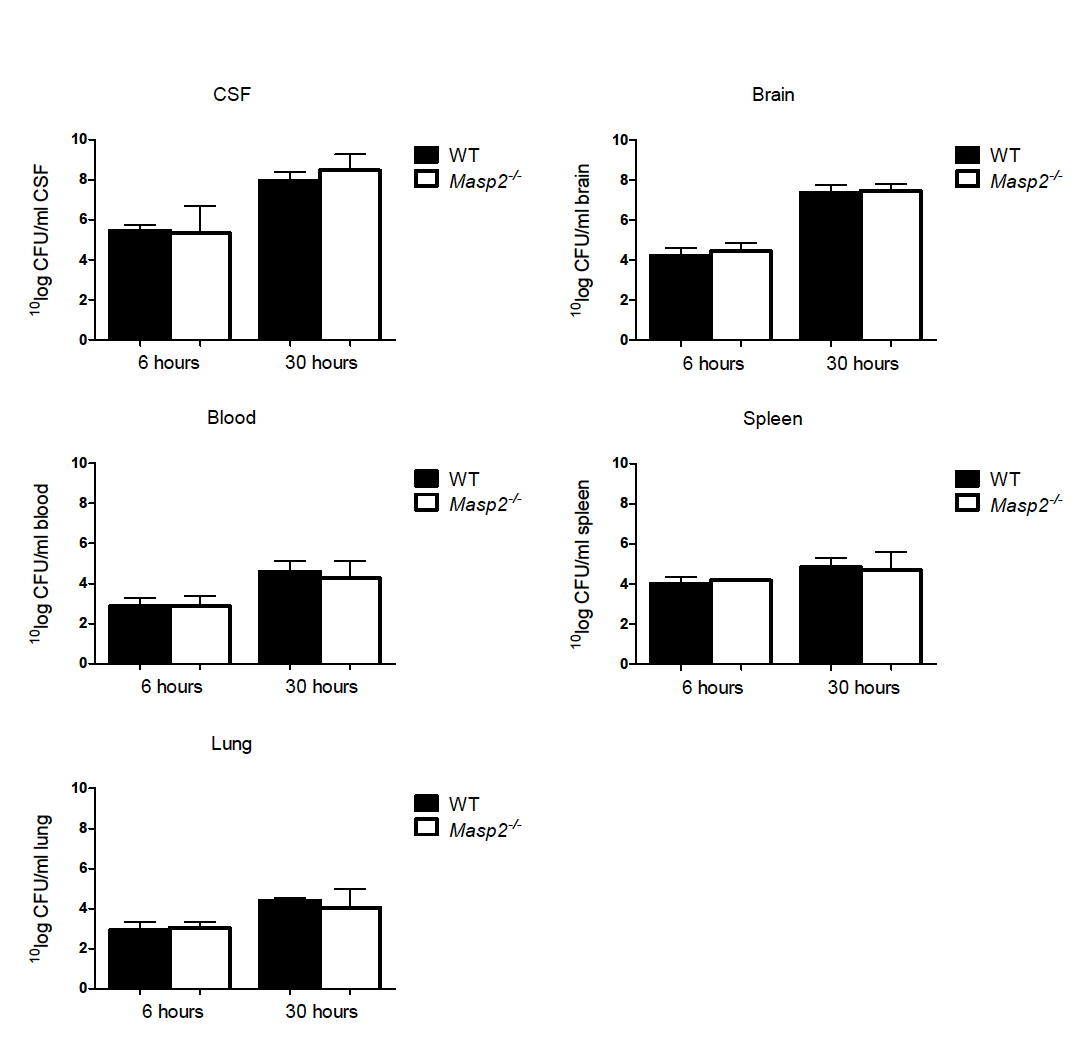


**Supplemental Figure 2.** Bacterial outgrowth in CSF, blood, brain, spleen and lung was similar between saline and Masp-2 antibody treated mice at 24 and 48 hours after infection. Data are given as medians and 75th quartile, P values were determined with the Mann-Whitney U test.


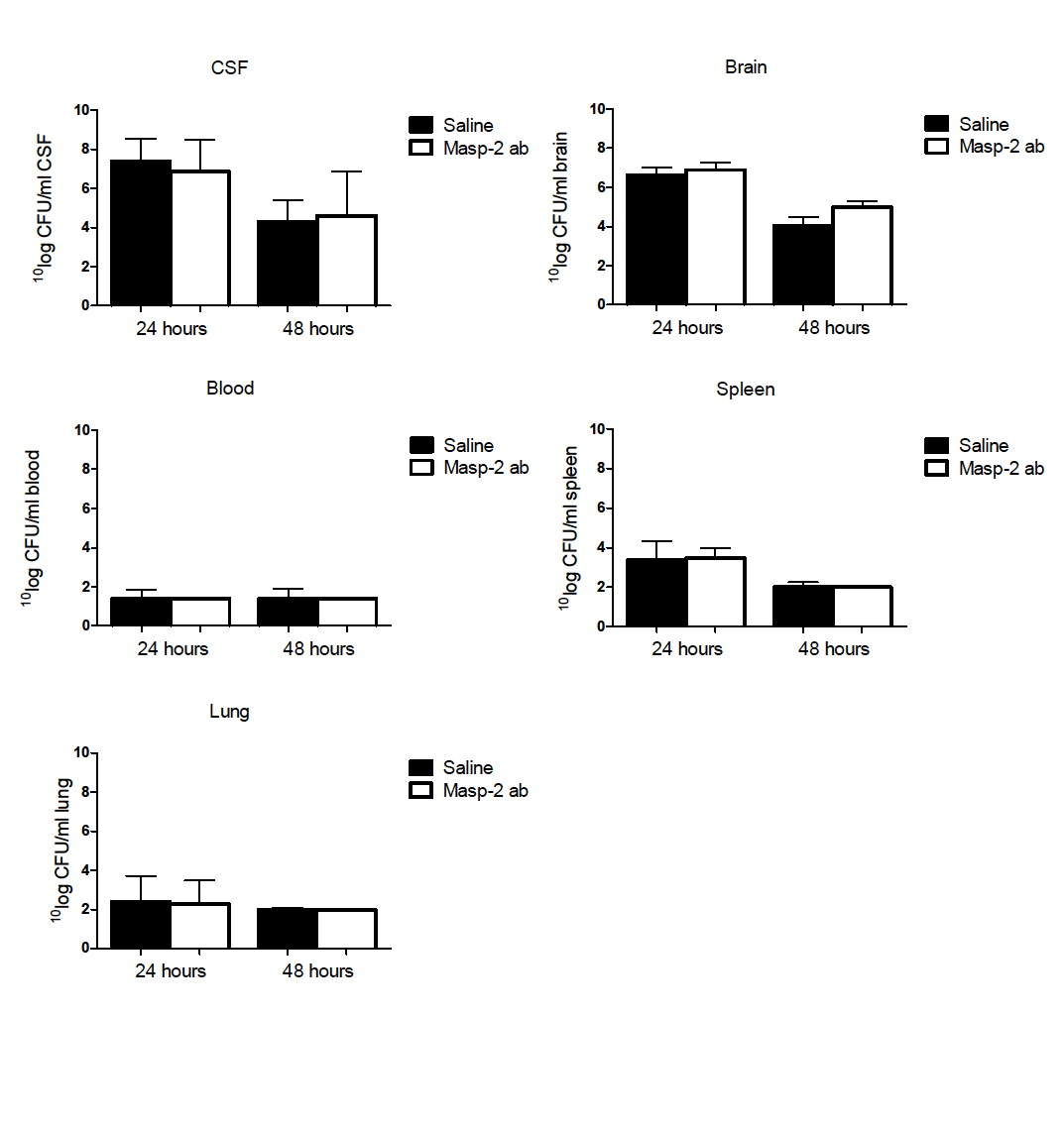


1. Anderson CA, Pettersson FH, Clarke GM, Cardon LR, Morris AP, Zondervan KT: **Data quality control in genetic case-control association studies**. *Nature protocols* 2010, **5**(9):1564-1573.

2. Purcell S, Neale B, Todd-Brown K, Thomas L, Ferreira MA, Bender D, Maller J, Sklar P, de Bakker PI, Daly MJ *et al*: **PLINK: a tool set for whole-genome association and population-based linkage analyses**. *American journal of human genetics* 2007, **81**(3):559-575.

3. Jennett B, Teasdale G, Braakman R, Minderhoud J, Knill-Jones R: **Predicting outcome in individual patients after severe head injury**. *Lancet* 1976, **1**(7968):1031-1034.
